# New Discrete Formulation for Reduced Population Balance Equation: An Illustration to Crystallization

**DOI:** 10.1007/s11095-022-03349-0

**Published:** 2022-08-09

**Authors:** Mehakpreet Singh, Gavin Walker

**Affiliations:** 1 Bernal Institute, Department of Chemical Sciences, University of Limerick, V94 T9PX Limerick, Ireland; 2 Bernal Institute, School of Engineering, University of Limerick, V94 T9PX Limerick, Ireland

**Keywords:** Aggregation, Integro-partial differential equation, Finite volume scheme, Cell average technique, Reduced model

## Abstract

In this paper, we focus on providing a discrete formulation for a reduced aggregation population balance equation. The new formulation is simpler, easier to code, and adaptable to any type of grid. The presented method is extended to address a mixed-suspension mixed-product removal (MSMPR) system where aggregation and nucleation are the primary mechanisms that affect particle characteristics (or distributions). The performance of the proposed formulation is checked and verified against the cell average technique using both gelling and non gelling kernels. The testing is carried out on two benchmarking applications, namely batch and MSMPR systems. The new technique is shown to be computationally less expensive (approximately 40%) and predict numerical results with higher precision even on a coarser grid. Even with a revised grid, the new approach tends to outperform the cell average technique while requiring less computational effort. Thus the new approach can be easily adapted to model the crystallization process arising in pharmaceutical sciences and chemical engineering.

## Introduction

In the experimental and quantitative analysis of disperse phase population dynamics, population balance equations (PBEs) have become an effective and efficient method for tracking the tracer mass. Various researchers have used tracer experiments to derive agglomeration kinetics parameters and breakage rate, as well as the age of the granules [10, 27]. Industrial applications such as sprayed fluidized bed granulator [9, 15] and twin-screw wet granulator [11, 12, 17, 43] in which multiple particle properties (size, shape, porosity and tracer mass) are required to describe the quality of the granules [13, 14, 30]. Using the application of the high shear granulation, Pearson *et al*. [27] conducted a study to track the tracer mass corresponds to a breakage process. Later, Hounslow *et al*. [8] developed a modeling approach for tracking the tracer mass changed due to aggregation and breakage processes which takes place in the high shear granulation. Hounslow *et al*. [8] idea is based on tracking the two internal properties of the particles of the system. They addressed a simple mathematical reduction of the complete two dimensional PBE into two different one-dimensional PBE’s, one that accounts for the granule size distribution (GSD) and the other for a tracer mass distribution (TMD). They also developed a new numerical method to solve these PBE’s, however, is computationally expensive due to its complex formulation.

### Population Balance Equation

A two-dimensional particle property distribution is defined as *g*(*t*, *v*, *x*) having properties $v,x > 0$ at time $t \ge 0$, that is, the number of particles in the infinitesimal range $[v, v+dv]\times [x, x+dx]$ at any time *t* is given by $g(t,v,x)\,dv\,dx$. A two dimensional aggregation PBE [19, 45] in a well mixed system can be written as

1 $$\begin{aligned} \frac{\partial g(t,v,x)}{\partial t}= & {} \frac{1}{2}\int _0^v \int _{\max (0, x-v+\eta )}^{\min (x, \eta )} \hat{\beta }(t,v-\eta , \eta , x-\theta , \theta )g(t, v-\eta , x-\theta ) g(t, \eta , \theta ) d \theta d \eta \nonumber \\&- \int _0^\infty \int _0^\eta \hat{\beta }(t, v, \eta , x, \theta ) g(t, v, x) g(t, \eta , \theta ) d \theta d \eta . \end{aligned}$$

supplemented with an initial condition

2 $$\begin{aligned} g(0,v,x)=g_0(v,x). \end{aligned}$$

The first integral on the RHS of Eq. (1) describes the birth of particles with properties (*v*, *x*) due to the aggregation of particles having properties $(v-\eta , x-\theta )$ and $(\eta , \theta )$. Similarly, the second integral provides the information of the omission of particles (*v*, *x*) that undergo coalescence with $(\eta , \theta )$. The aggregation kernel $\beta (t, v, \eta , x, \theta )$ represents the kinetics of two particles with attributes (*v*, *x*) and $(\eta ,\theta )$ colliding successfully. It is a non-negative function ($\beta (t, v, \eta , x, \theta )>0$) that is symmetric in terms of its property arguments. The aggregation kernel can be written in the form $\beta = \beta _0(t) \beta ^*(v, \eta , x, \theta )$. For the current study, time independent kernels are considered, however, the proposed approach can be implemented for any kind of kernel.

### Reduced Model

Many authors have proposed various exact solutions [4, 5, 16] and numerical techniques in order to solve the complete two dimensional original PBE (1). Those numerical methods involve cell average techniques [18, 33, 34, 39], fixed pivot techniques [48], stochastic methods [2, 25, 26] and finite volume schemes [6, 29, 32, 36, 38, 41, 44]. But due to non availability of the analytical technique to obtain the experimental data in complete two dimensions [30], various researcher used the approach of reduced model to track two properties of the granules independently. Recently, the reduced breakage model has been solved using the notion of highly efficient and accurate finite volume scheme [46]. In order to track two properties independently corresponding to the aggregation process, the 2-D PBE can be converted into two 1-D PBEs corresponding to the conventional number density *f*(*t*, *v*) [7] and mass of tracer within granules *m*(*t*, *v*). The number density *f*(*t*, *v*) can be obtained from *g*(*t*, *v*, *x*) by integrating over all possible tracer mass

3 $$\begin{aligned} \frac{\partial f(t,v)}{\partial t}= & {} \frac{1}{2}\int _{0}^v \beta (t,v-\eta ,\eta )f(t,v-\eta ) f(t,\eta ) d \eta \nonumber \\&- f(t,v) \int _{0}^\infty \beta (t,v,\eta ) f(t,\eta ) d \eta . \end{aligned}$$

Similarly, the mathematical expression to track the one dimensional PBE for tracer mass distribution is provided as follows:

4 $$\begin{aligned} \frac{\partial m(t,v)}{\partial t}= & {} \frac{1}{2}\int _{0}^v \beta (t,v-\eta ,\eta )m(t,v-\eta ) f(t,\eta ) d \eta \nonumber \\&- m(t,v) \int _{0}^\infty \beta (t,v,\eta ) f(t,\eta )d \eta . \end{aligned}$$

Equations (3) and (4) are classified as integro-partial differential equations which have to be solved numerically in order to track the granules size distribution and tracer mass distribution, respectively. The derivations of the above equations are provided in detail by Hounslow *et al*. [8] and Kumar *et al*. [19].

### Literature and Motivation

In the available literature, there are many analytical techniques available in the literature to track the experimental data for two properties of the granules independently (refer to [30] and references therein). In addition, many authors proposed different schemes to solve the aggregation PBE for granule size distribution including finite volume schemes [22, 31, 35, 37, 38, 40, 42], least square methods [3, 49], method of moments [1], stochastic methods [24], cell average techniques [21, 38] and fixed pivot techniques [20, 23, 48].

Now the question arises how one can develop a numerical method to approximate the set of reduced PBEs at moderate computational cost. Due to the complex nature of these equations, few numerical methods are available in the literature for solving a mass tracer aggregation PBE. The first numerical method to approximate the tracer PBE was developed by Hounslow *et al*. [8]. Later, Peglow *et al*. [28] modified the numerical approximation of the Hounslow *et al*. [8] to improve the accuracy of the numerical results. The main drawbacks of both numerical approaches are that they can only be implemented using a specific type of grid and size-independent kernel, which limits the applicability of both to granulation and crystallization processes. However, these real- life applications involve rigorous use of size dependent kernels specifically additive and multiplicative kernels as different volume particles are formed at different aggregation rates [11]. In 2006, Kumar *et al*. [19] presented a numerical method well known as cell average technique which overcome all issues of the existing methods. The idea of cell average is based on finding the average of all new born particles within the cell and then redistribute them to the neighbouring nodes in such a way that pre-chosen properties are exactly preserved. The major disadvantage of the cell average technique is recalculation of the birth term after the redistribution of particles properties to the neighbouring nodes which makes this method computational expensive. Another significant problem with this approach is that it predicts negative values for primary particles corresponding to size dependent kernels such as additive and multiplicative kernels. This limited the use of this numerical approach for solving real-life applications concerning granulation processes.

In this work, our aim is to propose a new framework based on the finite volume scheme for a mass tracer aggregation PBE whose mathematical formulation is simpler than the cell average technique and predict the numerical results more accurately and efficiently than the existing method. Moreover, the developed scheme is extended to solve a problem related to mixed-suspension mixed-product removal system in which aggregation and nucleation mechanisms are responsible for changing the particles properties. The convergence of the numerical results is discussed by approximating a mass tracer PBE on refined grids.

The rest of the article is organized as follows: Section 2 provides the detailed derivation of the finite volume scheme for solving a tracer mass distribution of aggregation PBE along with theoretical proof of volume conservation. Moreover, Section 3 is devoted to conduct the comparison of the numerical results for both batch and continuous system against exact solutions and the convergence is discussed for various grids. Further in Section 3.3 the discussion of the numerical results against exact solutions for continuous system using various grids are conducted. Finally, Section 4 provide the conclusions of the study.

## Numerical Method

In order to develop the numerical approximation for the tracer mass distribution (4), first it is important to fix the computational domain (upper limit $\infty$) to a finite number (say ${v}_{max}<\infty$) of the second integral in this equation. Thus the reduced model required to track the tracer mass distribution corresponding to the aggregation PBE can be reformulated as follows:

5 $$\begin{aligned} \frac{\partial m(t,v)}{\partial t}= & {} \int _{0}^v \beta (t,v-\eta ,\eta )m(t,v-\eta ) f(t,\eta ) d \eta \nonumber \\&- m(t,v) \int _{0}^{{v}_{max}} \beta (t,v,\eta ) f(t,\eta )d \eta , \end{aligned}$$

corresponding to a new initial condition

6 $$\begin{aligned} m(0,v) = m_0(v),~~v\in ~(0,{v}_{max}]. \end{aligned}$$

**Fig. 1:**
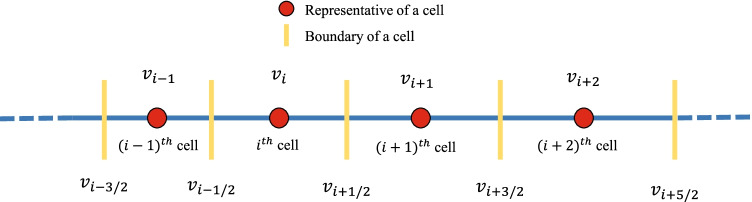
One dimensional domain discretization.

The numerical method is based on the assumption that particles within a grid cell are concentrated on its representatives. For the numerical methods, a finite one dimensional computational domain with upper limit, ${v}_{max}<\infty$, is divided into *I* number of smaller cells having $v_i$ as representative volume, for $i\in {1,2,...,I}$ (see Fig. 1). Now, define the grid points and the step size by

$$\begin{aligned} {v_{1/2}}=0,~{v}_i=\displaystyle \frac{{{ v}_{i-1/2} +{ v}_{i+1/2}}}{2}, ~ \Delta {v}_i={{ v}_{i+1/2} - {v}_{i-1/2}},~ v_{I+1/2}={v}_{max}. \end{aligned}$$

For the numerical approximation, let us first define the following set of indices

7 $$\begin{aligned} {\Upsilon }^i = \left\{ (j, k)\in \mathbb {N} \times \mathbb {N}: v_{i-1/2} < (v_j + v_k) \le v_{i+1/2} \right\} . \end{aligned}$$

**Fig. 2:**
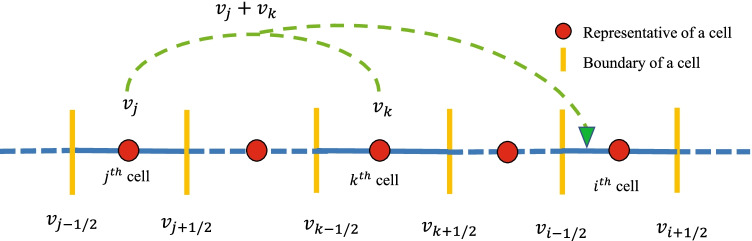
Representation of set ${\Upsilon }^i$.

Here $v_{i-1/2}$ and $v_{i+1/2}$ are the lower and upper ends of the *i*th cell, respectively. The set ${\Upsilon }^i$ denotes the sum of particles having properties $v_j$ and $v_k$ falls in a *i*th cell having properties $v_i$ (see Fig. 1). The graphical illustration of the ${\Upsilon }^i$ is shown in Fig. 2.

For $i\in {1,2,...,I}$, assume that $N_{i}$ and $m_{i}$ are the number of particles and total mass, respectively at time *t* in $i^{ {th}}$ cell which can be computed using the following expressions:

8 $$\begin{aligned} N_{i} = \int _ {v_{i-1/2}}^{ v_{i+1/2}} f(t, v) dv. \end{aligned}$$

and

9 $$\begin{aligned} m_{i}= \int _ {v_{i-1/2}}^{ v_{i+1/2}} vf(t, v) dv. \end{aligned}$$

The idea of the new approximation is to convert the original Eq. (5) of continuous integrals into set of ordinary differential equations by assuming that the point masses are concentrated on representatives, that is,

10 $$\begin{aligned} f(t,v) \approx \sum \limits _{k=1}^{I}N_k \delta (v-v_k), \end{aligned}$$

and

11 $$\begin{aligned} m(t,v) \approx \sum \limits _{k=1}^{I}m_k \delta (v-v_k). \end{aligned}$$

Integrating the original Eq. (5) over the boundaries of the $i^{th}$ cell leads to the following can be obtained:

12 $$\begin{aligned} \frac{dm_i(t)}{dt}= B_i(t)-D_i(t), \end{aligned}$$

where the birth and death terms are given by

13 $$\begin{aligned} B_{i}(t) = \int _ {v_{i-1/2}}^{ v_{i+1/2}} \int _0^v \beta (t, v-\eta , \eta ) m(t, v-\eta ) f(t, \eta )d \eta dv, \end{aligned}$$

and

14 $$\begin{aligned} D_{i}(t) = \int _ {v_{i-1/2}}^{ v_{i+1/2}} m(v) \int _{0}^{\infty } \beta (v,\eta ) N(\eta ) d \eta d v. \end{aligned}$$

### Simplification of Birth Tracer Term

For the simplification of notations, we omit the parameter *t* in our derivation and further assume $v_1 = 0$, the expression (13) can be written as follows

15 $$\begin{aligned} B_{i}(t)= & {} \int _{v_i}^{v_{i+1}} \sum \limits _{j=1}^{i-1} \int _{v_j}^{v_{j+1}} \beta (v-\eta ,\eta ) m(v-\eta ) f(\eta )d \eta d v \nonumber \\&+ \int _{v_i}^{v_{i+1}} \int _{v_i}^{v} \beta (v-\eta ,\eta ) m(v-\eta ) f(\eta )d \eta d v. \end{aligned}$$

Substituting $f(t,v) = \sum _{k=1}^I N_k \delta (v-v_k)$ and $m(v) = \sum _{k=1}^I m_k \delta (v-v_k)$ from Eqs. (10) and (11) in above Eq. (15), we get

16 $$\begin{aligned} B_{i}(t)= & {} \int _{v_i}^{v_{i+1}} \sum \limits _{j=1}^{i-1} \int _{v_j}^{v_{j+1}} \beta (v-\eta ,\eta ) \sum \limits _{k=1}^I [ m_k \delta (v-\eta -v_k)] \sum _{k=1}^I [N_k \delta (\eta -v_k)] d \eta d v \nonumber \\&+ \int _{v_i}^{v_{i+1}} \int _{v_i}^{v} \beta (v-\eta ,\eta ) \sum \limits _{k=1}^I [m_k\delta (v-\eta -v_k)] \sum \limits _{k=1}^I [N_k\delta (\eta -v_k)]d \eta d v. \end{aligned}$$

Using the notion of Dirac-delta distribution in the first integral and changing the order of integration in the second integral lead to the following:

17 $$\begin{aligned} B_{i}(t)= & {} \int _{v_i}^{v_{i+1}} \sum \limits _{j=1}^{i-1}\beta (v-v_j,v_j)\sum \limits _{k=1}^I [m_k \delta (v-v_j-v_k)] N_j d v \nonumber \\&+ \int _{v_i}^{v_{i+1}} \int _{\eta }^{v_{i+1}} \beta (v-\eta ,\eta ) \sum \limits _{k=1}^I [m_k\delta (v-\eta -v_k)] \sum \limits _{k=1}^I [N_k\delta (\eta -v_k)] d v d \eta . \end{aligned}$$

The above equation can be further simplified to

18 $$\begin{aligned} B_{i}(t)= & {} \sum \limits _{j=1}^{i-1} N_j \int _{v_i}^{v_{i+1}}\beta (v-v_j,v_j)\sum \limits _{k=1}^I [m_k \delta (v-v_j-v_k)] d v \nonumber \\&+ \int _{v_i}^{v_{i+1}} \beta (v-v_i,v_i) \sum \limits _{k=1}^I [m_k\delta (v-v_i-v_k)] N_i d v. \end{aligned}$$

Reapplying the definition of the Dirac-delta distribution in both the integrals finally gives

19 $$\begin{aligned} B_{i}(t) = \sum \limits _{j=1}^{i-1} N_j \sum \limits _{v_i\le (v_j+v_k)<v_{i+1}} \beta (v_k, v_j)m_k + \sum \limits _{(v_i + v_k)< v_{i+1}} \beta (v_k, v_i) m_k N_i . \end{aligned}$$

One can observe that for each term $m_j N_k$ in Eq. (19), there exist a term $m_k N_j$ except for $j=k$, then the Eq. (19) can be rewritten as

20 $$\begin{aligned} B_{i}(t) = \sum \limits _{(j, k) \in {\Upsilon }^i}\frac{1}{2} \beta (v_j, v_k)(m_j N_k + m_k N_j). \end{aligned}$$

### Simplification of Death Tracer Term

Equation (14), discretized up to $v_{I+1}$, can be rewritten as follows

21 $$\begin{aligned} D_{i}(t) = \int _{v_i}^{v_{i+1}} m(v) \sum \limits _{j=1}^{I}\int _{v_j}^{v_{j+1}}\beta (v,\eta )N(\eta )d \eta d v. \end{aligned}$$

Again using the application of Dirac-delta distribution, we get

22 $$\begin{aligned} D_{i}(t)= & {} \int _{v_i}^{v_{i+1}} \sum \limits _{k=0}^{I} [m_k \delta (v-v_k)] \sum \limits _{j=1}^{I}\int _{v_j}^{v_{j+1}}\beta (v,\eta )\sum \limits _{k=0}^{I} [N_k \delta (\eta -v_k)] d \eta d v \nonumber \\= & {} \int _{v_i}^{v_{i+1}} \sum \limits _{k=0}^{I} [m_k \delta (v-v_k)] \sum \limits _{j=1}^{I} \beta (v,v_j)N_j d v \nonumber \\= & {} \sum \limits _{j=1}^{I} N_j \int _{v_i}^{v_{i+1}} \sum \limits _{k=0}^{I} [m_k \delta (v-v_k)] \beta (v,v_j) d v \nonumber \\= & {} \sum \limits _{j=1}^{I} \beta (v_i,v_j)N_j m_i. \end{aligned}$$

Substituting the expressions (20) and (22) in equation (12), we define the finite volume scheme as:

23 $$\begin{aligned} \frac{dm_i(t)}{dt}= \sum \limits _{(j, k) \in {\Upsilon }^i} \frac{1}{2} \beta (v_j, v_k) (m_j N_k +m_k N_j)-\sum \limits _{j=1}^{I} \beta (v_i,v_j)N_j m_i. \end{aligned}$$

Further, divide the time domain as $t^{p+1}= t^{p}+\Delta t^p$ for $p\in \mathbb {N}$ and integrating over the time domain gives

24 $$\begin{aligned} m_i^{p+1} = m_i^p + \Delta t^p \left( \sum \limits _{(j, k) \in {\Upsilon }^i} \frac{1}{2}\beta ^p(v_j, v_k) (m_j^p N_k^p +m_k^p N_j^p)-\sum \limits _{j=1}^{I} \beta ^p(v_i,v_j)N_j^p m_i^p \right) . \end{aligned}$$

Here $m_i^p$ denotes the value of tracer mass at time $t^{p}$ in the $i^{th}$ cell. It can be observed that for the case of the cell average technique, the particle properties are distributed to the neighboring nodes, if the average of all particles properties after the aggregation do not fall on the representative in order to achieve the conservation of required moments. However, in case of the finite volume scheme, we need not any special treatment for conserving the mass conservation law (Theoretical proof is provided in Appendix A).

## Simulation Results and Discussion

This section is devoted to check the accuracy and efficiency of the newly developed finite volume scheme against the existing cell average technique [19] for different computational domains. In order to conduct the comparison intensively, two cases, namely, batch system and mixed-suspension mixed-product removal (MSMPR) system are considered. The monodisperse initial condition $f_0(v)=\delta (v-1)$ and the computation domain considered is $v_{i+1}=2^{1/p}v_{i}$ for $p=1,3~ \text {and}~ 5$ for the comparison. To compare the numerical results, it is necessary to define the degree of aggregation as follows:

25 $$\begin{aligned} I_{agg} = {\left\{ \begin{array}{ll} 1-\displaystyle \frac{\mu _0(t)}{\mu _0(0)}, &{} \text{ batch } \text{ system }, \\ 1-\displaystyle \frac{\mu _0(t)}{\mu _0^{\text {in}}(0)}, &{} \text{ continuous } \text{ system }. \end{array}\right. } \end{aligned}$$

Here $\mu _0(t)$ expresses the zeroth order moment (total number of particles ) at any time *t* and $\mu _0^{\text {in}}(0)$ is the zeroth order moment of the feed. The testing will be conducted for both batch and MSMPR systems using sum and product kernels. Smit *et al*. [47] have shown that the sum kernel shows gelling behavior for the continuous system and non gelling behavior for batch systems. However, the product kernels is classified as a gelling kernel for both batch and continuous systems. For the gelling kernels simulations are run till their gelation point. Gelation is a phase transition that occurs during the aggregation process where mass is lost from particles of finite volume and appears in particles of infinite volume instead [47]. Therefore, predicting the numerical solutions for these kernels are highly challenging. The integration of discrete form of TMD (12) is solved using MATLAB ODE15s solver. The numerical simulations are run on machine with specifications i5 7300U CPU with 2.70 GHz and 16 GB RAM.

### Simulations for Batch System Using Sum Kernel

The comparison is began by considering the additive kernel, $\beta (v,\eta )=\beta _0(v+\eta )$ where $\beta _0=1$. We compare the total number of primary particles ($N_{r,i}$) which represents *m*(*t*, *v*) in continuous MSPMPR system and is given by

26 $$\begin{aligned} N_{r,i}(t)=iN_i(t), \end{aligned}$$

where *i* is identified as the number of primary particles in the size of a granule *v*. Moreover, the mean volume size of the primary particle distribution is also calculated for the comparison using the following relation:

27 $$\begin{aligned} \bar{\chi }_{r}(t)=\displaystyle \frac{\sum _{i}N_{r,i}(t)}{\sum _{i}iN_i(t)}~ \forall ~ t. \end{aligned}$$

**Fig. 3:**
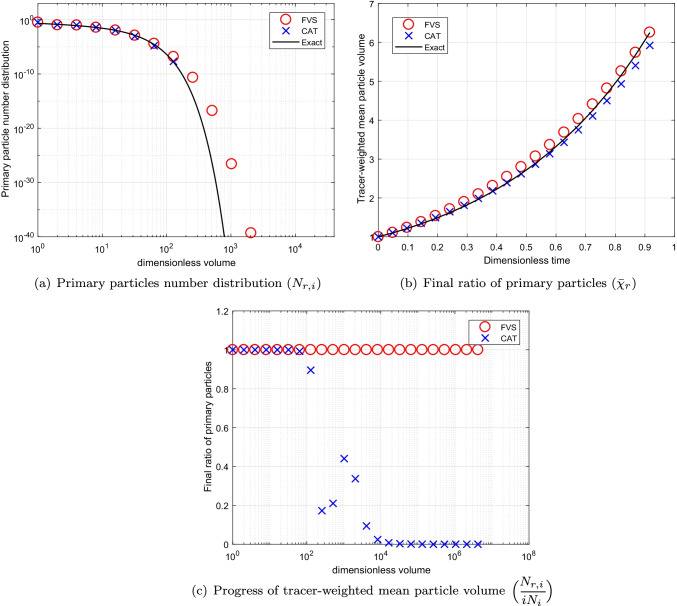
Numerical results using additive kernel with a geometric grid of 23 cells for a batch system.

For the additive kernel, the mean volume size of the primary particle distribution is given by $\bar{\chi }_{r}(t)= e^{2t}$. The degree of aggregation for this particular case is considered to be $I_{agg}=0.80$. The numerical results against exact results for this case are plotted in Fig. 3 for $p=1$. It can be seen that the primary particles number distribution predicted by the new scheme shows better results than the existing scheme as the existing scheme is not able to calculate the primary particles population for larger volumes. This is possibly due to the reason that for the case of the cell average technique, the particles take birth in the last cell leads to numerical error because of the distribution of the particles properties to the neighboring nodes which ends up in losing the volume from the system, however, this is not possible in the new scheme as no particles properties to the neighboring nodes are required to be distributed. However, the new scheme predicts the larger volume population of primary particles with less precision (see Fig. 3(a)). Moreover, the final ratio of primary particles and progress of tracer-weighted mean particle volume plotted in Fig. 3(b) and (c) reveal that the new scheme is highly accurate than the existing scheme.

**Fig. 4:**
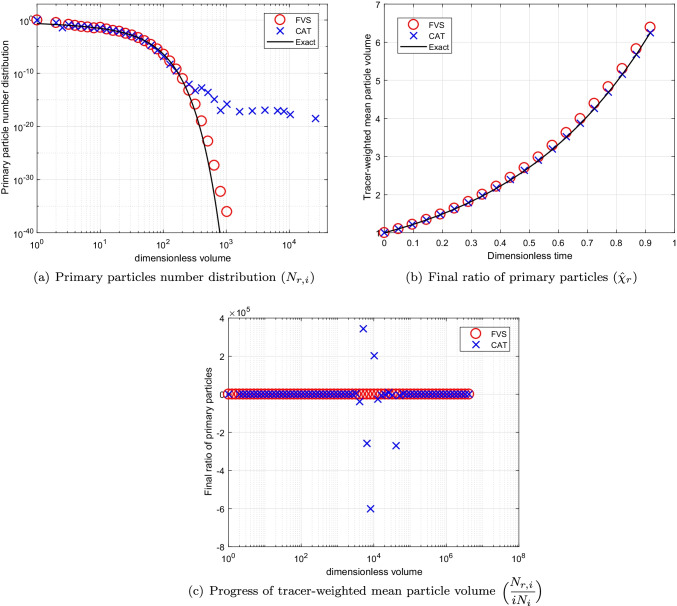
Numerical results using additive kernel with a geometric grid of 67 cells for a batch system.

**Fig. 5:**
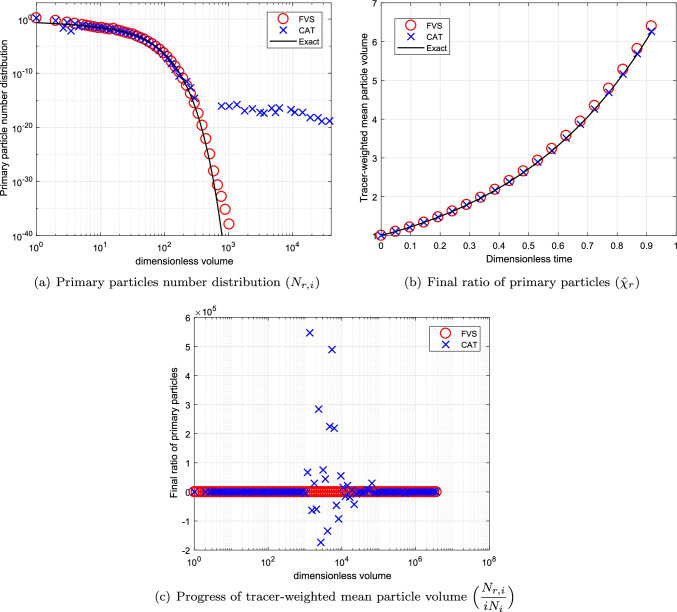
Numerical results using additive kernel with a geometric grid of 110 cells for a batch system.

To see the convergence of the numerical results towards the exact results, we compare the numerical results obtained using refined grids corresponding to $p=3~ \text {and}~ 5$ in Figs. 4 and 5, respectively. Figures conclude that the results for both schemes improve to larger extend, however, still the new scheme performs better than the existing scheme (refer to Figs. 4(a), (b), 5(a) and (b)). Additionally, it can also be observed that in the case of the cell average technique, the negative values for the final ratio of primary particles increase to large extent as more refined grids are considered for obtaining the results as demonstrated in Figs. 4(c) and 5c. Whereas the new scheme establishes very stable results for even refined grids. In terms of computational CPU time, the new scheme is more efficient in obtaining the numerical results than the existing scheme (See Table I).

**Table I:** Computational Time Using Additive Kernel for a Batch System

Cells	CPU time	CPU time
	CAT	FVS
23	0.69	0.48
67	2.46	1.89
110	4.89	3.39

### Simulation for Batch System Using Product Kernel

Next we compare the numerical results for the product kernel ($\beta (v,\eta )=\beta _0(v\eta )$) using various size computational domains. For this kernel, the analytical mean volume size of the primary particle distribution is given by $\bar{\chi }_{r}(t)= \displaystyle \frac{1}{1-t}~ \text {for}~ 0\le t <1$. The simulations are run for the degree of aggregation $I_{agg}=0.50$.

**Fig. 6:**
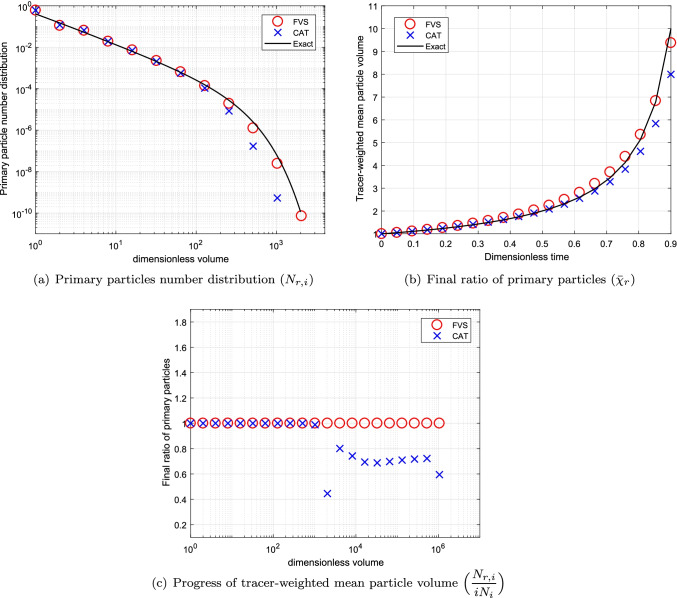
Numerical results using product kernel with a geometric grid of 21 cells for a batch system.

The numerical results for both numerical methods are plotted in Fig. 6 for $p=1$. Alike the previous case, the numerical results for a primary particles number distribution computed by the new scheme shows much higher accuracy than the existing scheme (see Fig. 6(a)). Moreover, the final ratio of primary particles estimated by the new scheme overlap with the exact result whereas the existing scheme shows under prediction from the exact result for as concluded in Fig. 6(b). In addition, the new scheme predicted the tracer-weighted mean particle volume with higher precision than the existing scheme. Even for this case, the existing scheme gives negative values for this result as shown in Fig. 6(c).

**Fig. 7:**
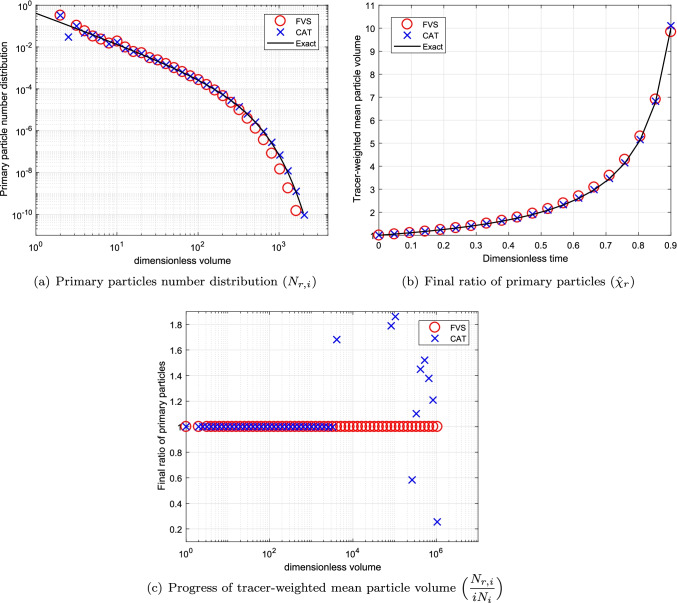
Numerical results using product kernel with a geometric grid of 61 cells for a batch system.

To check the convergence of the results for the refined grids, the numerical results are plotted in Fig. 7 for computational grids corresponding to $p=3$, respectively. It can be observed that both schemes acquire same accuracy for both primary particles number distribution as well as final ratio of primary particles as demonstrated in Fig. 7(a) and (b). In addition, the tracer-weighted mean particle volume determined by the new scheme shows more accuracy than the existing scheme for computational domain obtained for $p=3$ (see Fig. 7(c)). The efficiency of both numerical methods in terms of CPU time is compared and listed in Table II. One can observe that the new scheme obtained the numerical results by consuming 50% lesser time than the existing scheme for different grids.

**Table II:** Computational Time Using Product Kernel for a Batch System

Cells	CPU Time	CPU Time
	CAT	FVS
21	1.42	0.80
61	5.19	3.67
101	14.66	7.26

### Aggregation and Nucleation in Continuous MSMPR

In this section, we consider a problem formulated by [10] in order to test the applicability of the newly developed discrete formulation. In this particular problem, a monodisperse tracer was added to a well-mixed continuous particle process that was initially assumed to be in steady state. In this case, two mechanisms namely aggregation and nucleation are effecting the properties of the particles. The population balance equation required to model this system are given as follows:

28 $$\begin{aligned} \frac{\partial f(t,v)}{\partial t}= & {} \int _{0}^v \beta (t,v-\eta ,\eta )f(t,v-\eta ) f(t,\eta ) d \eta - f(t,v)\int _{0}^{{v}_{max}} \beta (t,v,\eta ) f(t,\eta )d \eta \nonumber \\&+B_0\delta (v)-\frac{f(t,v)}{\tau },~~ f(t,0^-) = 0, ~~\frac{\partial f(t,v)}{\partial t}\Big |_{t=0}=0, \end{aligned}$$

and

29 $$\begin{aligned} \frac{\partial m(t,v)}{\partial t}= & {} \int _{0}^v \beta (t,v-\eta ,\eta )m(t,v-\eta ) f(t,\eta ) d \eta - m(t,v)\int _{0}^{{v}_{max}} \beta (t,v,\eta ) f(t,\eta )d \eta \nonumber \\&+B_0\delta (v)-\frac{m(t,v)}{\tau }, ~~ m(t,0^-) = 0, ~~\frac{\partial m(t,v)}{\partial t}\Big |_{t=0}=\delta (v-v_0). \end{aligned}$$

The exact results for decay of total tracer mass and tracer-weighted mean particle volume corresponding to both additive and product kernels is provided by Ilievski and Hounslow [10]. The mean mass for this continuous system can be calculated using

30 $$\begin{aligned} \hat{\lambda }_T(t) = \frac{\int _{0}^{\infty }vm(t,v)dv}{\int _{0}^{\infty }m(t,v)dv}. \end{aligned}$$

Here $T=t/\tau$. For running the simulation similar grid is considered as previous cases and $B_0=\tau =v_0$ given in Table IV denote the nucleation constant, dimensionless time and initial average volume, respectively. To enhance the comparison, the relative error in the mean volume is also calculated for different size grids using the relation:

31 $$\begin{aligned} {\text {Relative~Error}} =\left| \left| \frac{ \displaystyle \frac{\mu _1^{ana}*v}{\mu _1^{ana}}- \displaystyle \frac{\mu _1^{num}*v}{\mu _1^{num}}}{\displaystyle \frac{\mu _1^{ana}*v}{\mu _1^{ana}}}\right| \right| , \end{aligned}$$

where $\mu _1^{ana}$ and $\mu _1^{num}$ denote the analytical and numerical values of tracer mass in the system, respectively. The exact results tracer-weighted mean particle volume are listed in Table III.

**Table III:** Exact Solutions of Tracer-Weighted Mean Particle Volume

Cases	$\beta (v,\eta )$	${\hat{\lambda }_T}/{v_0}$
1.	$\beta _0(v+\eta )$	$\displaystyle \frac{1-I_{agg}}{3I_{agg}-1}+\displaystyle \frac{4I_{agg}-2}{(3I_{agg}-1)} \exp \left( \frac{t}{\tau }\displaystyle \frac{2I_{agg}}{(1-I_{agg})}\right)$
2.	$\beta _0(v\times \eta )$	$\exp \left( \displaystyle \frac{t}{\tau }\displaystyle \frac{1-\sqrt{1-8I_{agg}}}{2}\right)$

**Table IV:** Parameter Values for Running the Numerical Simulations

Parameters	Values
$\beta _0,~B_0,~\tau ,~v_0$	1
$v_{max}$	500 (for sum kernel)
$v_{max}$	100 (for multiplicative kernel)
Number of grids (*I*)	23, 67 and 110 for $p=1,~3~ \text{ and }~ 5$
$m_0(v)$	Initial mass density
$f_0(v)$	Initial number density

#### Simulations for Continuous System Using Sum Kernel

**Fig. 8:**
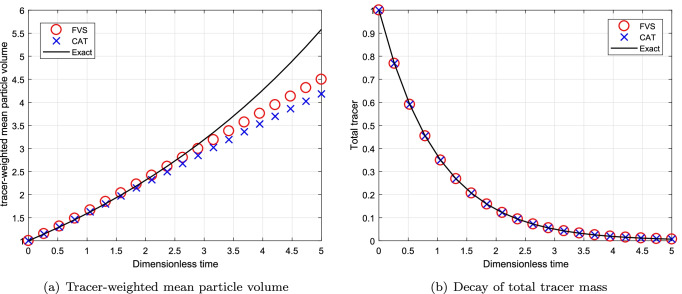
Numerical results using additive kernel with a geometric grid of 23 cells for a continuous system.

**Fig. 9:**
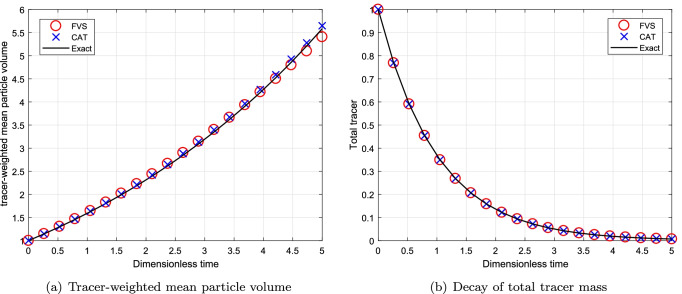
Numerical results using additive kernel with a geometric grid of 67 cells for a continuous system.

To begin the comparison for continuous MSMPR system, we first consider the additive kernel. The simulations are run till degree of aggregation $I_{agg}=1/6$. The decay of total volume of tracer for this case is $m_T = m_0\exp (-T)$ where $T=t/\tau$. Figure 8 shows the numerical results for a computational domain $v_{i+1}=2^{1/p}v_{i}$ for $p=1$. The decay of total tracer mass predicted more accurately by the new formulation than the existing scheme. However, both numerical schemes predict the tracer-weighted mean particle volume same accuracy and match well with the exact result. Moreover, the results obtained for decay of total volume of tracer using a computational domain $v_{i+1}=2^{1/p}v_{i}$ for $p=3$ is demonstrated in Fig. 9. One can observed that the accuracy shown by both numerical schemes is equal on a refined grid and converge to the exact result (refer to Figs. 8(a), (b), 9(a) and (b)).

The relative errors in decay of total tracer mass for different grids are provided in Table V. Table shows that the new scheme computed the numerical results with lesser errors whereas the existing scheme predicted these results with higher errors. Similarly, the CPU time taken by the new scheme and existing scheme is listed in Table VI and reveals that the new scheme obtained the numerical results by consuming nearly 50% lesser CPU time than the existing scheme.

**Table V:** Relative Error in the Mean Volume for Additive Kernel for a Continuous System

Cells	CAT	FVS
23	0.1715	0.1243
67	0.0179	0.0128

**Table VI:** Computational Time for Additive Kernel for a Continuous System

Cells	CPU Time	CPU Time
	CAT	FVS
23	1.21	0.54
67	2.92	1.71

#### Simulations for Continuous System Using Multiplicative Kernel

**Fig. 10:**
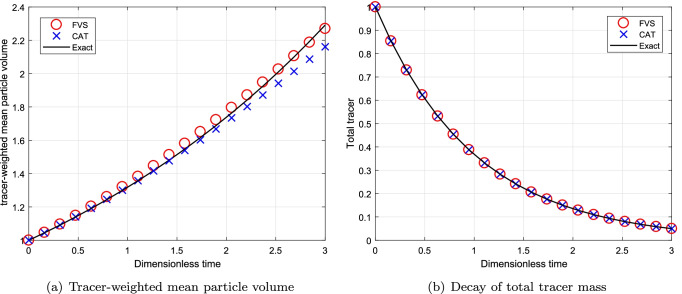
Numerical results using multiplicative kernel with a geometric grid of 21 cells for a continuous system.

**Fig. 11:**
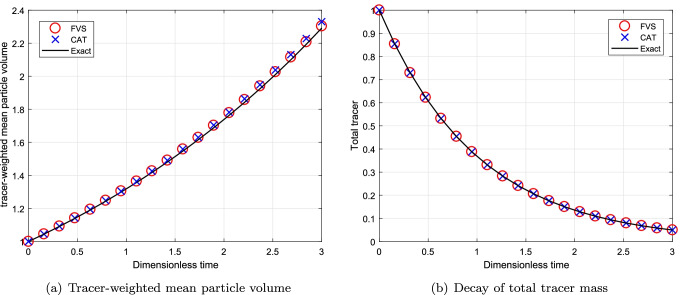
Numerical results using multiplicative kernel with a geometric grid of 61 cells for a continuous system.

Next, the numerical comparison is conducted for a MSMPR System using multiplicative kernel $\beta (v, \eta )=\beta _0(v\times \eta )$. The decay of total volume of tracer for multiplicative kernel is $m_T = m_0\exp (-T)$ where $T=t/\tau$ and thhe degree of aggregation is considered to be $I_{agg}=1/10$. The comparison of different results corresponding to the computational domain $v_{i+1}=2^{1/p}v_{i}$ for $p=1$ is shown in Fig. 10. In addition, to check the convergence of numerical results, we plotted the different numerical results for a computational domain $v_{i+1}=2^{1/p}v_{i}$ for $p=3$ in Fig. 11. The use of refined grid for obtaining the numerical results of tracer-weighted mean particle volume and decay of total tracer mass lead to converge to exact results. One can see that the tracer-weighted mean particle volume predicted by the new scheme is in better agreement with the exact result than the existing scheme, that is, the new scheme overlaps with the exact results whereas the existing scheme under predicted this result (refer to Figs. 10(a) and 11(a)). However, the numerical results for decay of total tracer mass is equally well obtained by both schemes as demonstrated in Figs. 10(b) and 11(b).

Moreover, the relative errors in decay of total tracer mass predicted by both numerical methods shows that the new scheme is 50% more accurate than the existing scheme for both grids (refer to Table VII). Similar to the previous cases, the new scheme takes lesser CPU time to compute the numerical results for this case as well as shown in Table VIII.

**Table VII:** Relative Error in the Mean Volume for Multiplicative Kernel for a Continuous System

Cells	CAT	FVS
21	0.0299	0.0142
61	0.0113	0.0068

**Table VIII:** Computational Time(s) for Multiplicative Kernel for a Continuous System

Cells	CPU Time	CPU Time
	CAT	FVS
21	1.00	0.51
61	2.72	1.26

## Conclusions

In this article, a discrete formulation based on finite volume scheme is developed for approximating the tracer mass distribution corresponding to pure aggregation and simultaneous aggregation-nucleation population balance equations. It is shown that the new formulation is very simple and can be implementing to any kind of aggregation kernel in contrast to the formulation developed by Hounslow *et al*. [8]. The accuracy and efficiency of the newly developed formulation is compared with the cell average technique for different aggregations kernel using different size grids. It is shown that the newly developed formulation shows better accuracy than the cell average technique for a coarser grid for several problems and leads to same accuracy for both schemes for a refined grid. In addition, it is also demonstrated that the newly developed discrete formulation is more efficient than the cell average technique, that is, the new scheme consumes lesser CPU time for obtaining the numerical results.

We finally conclude that the new scheme is more beneficial for solving problems related to the crystallization, twin screw granulation and sprayed fluidized bed granulation due to its simple mathematical formulation and accuracy.

## Data Availability

There is no research data associated with this paper.

## Appendix A: Mass Conservation Law

For any numerical method, the important criteria is that it satisfies the mass conservative law. Any numerical method follows the the mass conservative law if it satisfies the condition given below:

$$\begin{aligned} \sum \limits _{i=1}^{I}m_i(t^{p+1}) = \sum \limits _{i=1}^{I}m_i(t^{p}). \end{aligned}$$

### Proposition 1

The numerical formulation (24) does hold the mass conservation law, that is, the first order moment is conserved.

### Proof

By taking summation over all *i* of discrete formulation provided in Eq. (24), the right-hand side can be evaluated to

$$\begin{aligned} m_i(t^{p+1}) = m_i(t^{p}) + \Delta t^p T, \end{aligned}$$

where

A.1 $$\begin{aligned} T = \sum \limits _{(j, k) \in {\Upsilon }^i} \frac{1}{2}\beta ^p(v_j, v_k) (m_j^p N_k^p +m_k^p N_j^p)-\sum \limits _{j=1}^{I} \beta ^p(v_i,v_j)N_j^p m_i^p. \end{aligned}$$

Using relation $N_i=m_i/v_i$, the above equation can also be written as

A.2 $$\begin{aligned} T = \frac{1}{2} \sum \limits _{i=1}^I\sum \limits _{(j, k) \in {\Upsilon }^i}\beta ^p(v_j,v_k) (v_j+v_k)\frac{m_j^p}{v_j} \frac{m_k^p}{v_k} - \sum \limits _{i=1}^I\sum \limits _{j=1}^I \beta ^p(v_i,v_j) m_i^p \frac{m_j^p}{v_j} . \end{aligned}$$

On multiplying and dividing the second term of above equation by $u_i$, it gives

A.3 $$\begin{aligned} T= & {} \frac{1}{2} \sum \limits _{i=1}^I\sum \limits _{(j, k) \in {\Upsilon }^i} \beta ^p(v_j,v_k) v_k\frac{m_j^p}{v_j} \frac{m_k^p}{v_k} +\frac{1}{2} \sum \limits _{i=1}^I\sum \limits _{(j, k) \in {\Upsilon }^i} \beta ^p(v_j,v_k) v_k\frac{m_j^p}{v_j} \frac{m_k^p}{u_k} \nonumber \\&- \sum \limits _{i=1}^I\sum \limits _{j=1}^I \beta ^p(v_i,v_j) u_i \frac{m_i^p}{v_i} \frac{m_j^p}{v_j} . \end{aligned}$$

Using the symmetry of the aggregation kernel will lead to the following simplification

A.4 $$\begin{aligned} T = 2\frac{1}{2} \sum \limits _{i=1}^I\sum \limits _{j=1}^I \beta ^p(v_i,v_j) v_i \frac{m_i^p}{v_i} \frac{m_j^p}{v_j}- \sum \limits _{i=1}^I\sum \limits _{j=1}^I \beta ^p(v_i,v_j) v_i \frac{m_i^p}{v_i} \frac{m_j^p}{v_j} . \end{aligned}$$

This implies $T=0$. Hence the formulation (24) holds the mass conservation law.

$$\square$$
